# Conservative Surgery

**Published:** 1862-01

**Authors:** 


Conservative Surgery.—In the summer of 1846 (I was then living in Leicester-place) a servant drove up to my door in a street cabriolet—she had come from Wellington street, Strand—and requested me to return with her to see her master, a gentleman of more than seventy years of age, who had fallen down and seriously injured himself. Anticipating some occasion for strapping, lint, &c., I stayed long enough to provide myself with them, and then returned with her as quickly as the crowded state of the thoroughfares would permit. On arriving at the house I was hurried up to the drawing-room, where I was met by my patient, who, holding a handkerchief to his face, said, "Doctor, I have cut my nose off." I was at the moment rather incredulous, but his daughter soon removed all doubt as to the nature and extent of the injury by showing me the separated portion, which she had picked up from the floor. It was black, cold, and covered with grit and dirt. On examining the wound, I found that the whole of the fleshy end of the nose, together with the alæ and septum, were clean cut away, and the white end of the cartilage exposed. The upper lip was divided transversely throughout its whole extent, and hung down over the mouth. It appeared that this gentleman, on going up stairs, had stumbled near the upper step, and, trying to recover himself, had fallen forwards against a wooden flap placed at the drawing-room door, the sharp edge of which had come in contact with his nose, first compressing it and then separating it from the face.

For a moment I hesitated what to do, but thinking the separated part would be as good a dressing as any other to the exposed surface, and that the patient's hope (though I had none) of its re-union would give time for him to reconcile himself to his ultimate loss, I determined on re-adjusting it. This was easily enough done. The grit was wiped from it, and being carefully replaced, it was retained *in situ* by adhesive straps. The edges of the wound of the lip were brought together, and kept so by similar means.

On calling the next day, I observed that the end of the nose—which I had purposely left exposed—had lost the deep-black color that it had when replaced, and bore evident signs of circulation going on in it. There was no discharge from the wound. On the next day, appearances were the same; and on the third, the dressings were removed, when I was as much astonished as gratified to find that union had taken place throughout the whole extent, and the scar that was left was scarcely perceptible. The lip had also united.

Now the time which had elapsed between the separation and the re-adjustment of the divided parts could not have been less than three quarters of an hour.—*J. Nichols, M. R. C. P., London.*

In connection with this case of conservative surgery, we translate from the *Campagne de Russie* of Baron Larrey, the following:

A Russian colonel had received from one of our troops a sabre-cut which which had completely separated the nose from its base. The sabre striking obliquely, had cut through the superior maxillary bones, completely down to the mouth through the hard palate, so that the nose and anterior portion of the maxillary bone fell over upon the chin, attached and suspended only by a narrow strip of flesh at the corners of the mouth. We could see, on one side, the whole extent of the nasal fossæ and the cavity of the mouth, without its row of teeth; on the other, the flap, consisting of the whole nose, the upper lip and palatine arch, lying over on the chin. One of my assistants, having found the flap cold and attached only by the two points which I have mentioned, was going to remove it and dress the wound when I arrived. I put aside the surgeon's scissors, and after examining the flap prepared to replace it. Removing the clots from the nasal fossæ with some difficulty, I then detached that portion of the palatine arch which was attached to the flap, which included the anterior half of the upper alveolar arch. The line of the wound on one side was between the canine and the first bicuspid teeth; on the other, between the two bicuspids. I removed also from the flap many pieces of the nasal bones and processes of the maxillary bone. I then restored the nose and lip to their place, retaining them with the interrupted suture, beginning at the bridge of the nose, and descending successively on the two sides, until the edges were united by ten parallel sutures, and covered the whole wound with a light compress with salt water. I introduced into the nostrils two pieces of large-sized gum-elastic sound, to preserve their shape and size—having united the sounds at their outer end by a thread to keep them in place. Graduated compresses were placed on the sides of the nose, retained by a bandage, which completed the dressing.

I had the satisfaction of learning, on my return from Moscow, that this officer had entirely recovered, and without any deformity. The cure is remarkable from the severity of the wound and the scarcity of vessels which keep up communication between the flap and the integuments of the face. Vitality was restored in the nose, and its point of union was exact and perfect.—*Berkshire Medical Journal.*

